# Comparative Evaluation of Different Teeth Whitening Agents on Extrinsic Stains: An In-Vitro Interventional Study

**DOI:** 10.4317/jced.64122

**Published:** 2026-06-29

**Authors:** Meghna Bhargava, Pallavi Vashisth, Sathyajith Naik N., Shivangi Sharma, Resham Payak, Shilpa Kumari

**Affiliations:** 1 PG student. Department of Pediatric and Preventive dentistry, Institute of Dental Sciences, Bareilly; 2 Professor. Department of Pediatric and Preventive dentistry, Institute of Dental Sciences, Bareilly; 3 Principal, Professor and Head. Department of Pediatric and Preventive dentistry, Institute of Dental Sciences, Bareilly; 4 PG student. Department of Pediatric and Preventive dentistry, Institute of Dental Sciences, Bareilly

## Abstract

**Background:**

Tooth discolouration caused by extrinsic factors such as medications and dietary chromogens is a common aesthetic concern. While various whitening agents are available, their effectiveness may vary depending on the type of stain and formulation. Natural alternatives are gaining attention due to their potential safety and cost-effectiveness.

**Materials and Methods:**

60 extracted permanent maxillary incisors were randomly divided into two main groups based on staining agents: iron medication and pomegranate juice (n = 30 each). Each group was further subdivided into three subgroups (n = 10) based on the whitening modality used: whitening strips, whitening gel, and a customized herbal toothpaste containing banana peel extract. Staining procedures were carried out for 15 days, followed by a 14-day whitening protocol. Colour measurements were recorded at baseline, post-staining, and post-whitening using a spectrophotometer based on the CIE Lab* system. Colour change (E) was calculated and statistically analysed using paired t-test, unpaired t-test, and ANOVA.

**Results:**

Both staining agents produced significant tooth discolouration, with iron causing greater staining than pomegranate juice. All whitening modalities resulted in reduction of E values; however, the herbal formulation showed the highest improvement (p value = 0.00000878*), followed by whitening strips and whitening gel. Pomegranate-induced stains demonstrated greater reversibility compared to iron-induced stains. Statistical analysis revealed significant intra-group and inter-group differences (p &lt; 0.05).

**Conclusions:**

The effectiveness of tooth-whitening agents is influenced by both stain type and treatment modality. The banana peel-based herbal formulation demonstrated promising whitening potential and may serve as a safe and effective alternative to conventional agents. Further in vivo studies are recommended to confirm these findings.

## Introduction

Tooth discolouration, whether extrinsic or intrinsic, compromises dental aesthetics and may adversely affect a patient's self-confidence and overall appearance (1). Beyond its physical manifestation, it has a significant psychosocial impact, influencing self-esteem, social interactions, and oral health-related quality of life (2). Consequently, the demand for safe and effective tooth-whitening procedures has increased substantially in contemporary dental practice. Tooth discolouration is broadly classified into extrinsic and intrinsic types. Extrinsic stains result from the deposition of chromogenic substances on the tooth surface, whereas intrinsic discolouration arises from structural alterations within enamel or dentin due to systemic or developmental factors (1). Among extrinsic causes, medication-induced staining is of particular clinical relevance. Iron supplements, commonly prescribed for iron deficiency anemia, are frequently associated with noticeable tooth discoloration (3). Iron deficiency anemia remains a major global health concern, especially among young children, where iron supplementation is routinely administered as syrups or drops (4). Prolonged use has been linked to dark staining, attributed to the formation of ferric sulfide when iron ions interact with hydrogen sulfide produced by oral bacteria, resulting in insoluble iron deposits on enamel surfaces (5). Pigmented beverages such as pomegranate juice contain acids and are high in dietary fiber and nutrients such vitamins (C, A, and folic acid), minerals (potassium, magnesium, and copper), phenolic compounds, alkaloids, triterpenes, and sterols. The acidic pH and phenolic compounds lowers the pH around the enamel, destroys the hydroxyapatite structure, causing loss of enamel surface structure leading to gradual colour changes with repeated exposure (6). The increasing prevalence of tooth discolouration has driven the evolution of whitening techniques, ranging from conventional prophylaxis to advanced bleaching and restorative procedures (7). Professional bleaching typically employs high concentrations of hydrogen or carbamide peroxide, which, although effective, may cause adverse effects such as tooth sensitivity and gingival irritation (4). To overcome these limitations, at-home whitening systems have gained popularity due to their convenience and affordability. These include whitening strips, paint-on gels, and tray-based systems using lower concentrations of peroxide-based agents that penetrate enamel to achieve bleaching (8 , 9). Recently, there has been growing interest in natural and herbal whitening agents due to their perceived safety and minimal side effects. Substances such as banana peel have shown potential owing to their mineral content and bioactive compounds, which may facilitate stain removal through mild abrasive and chemical interactions. (10 , 11) Objective evaluation of tooth colour is essential in assessing whitening efficacy, with spectrophotometry providing reliable and reproducible measurements (2). Current literature lacks well-controlled comparative studies assessing the effectiveness of natural versus commercial whitening agents on stains induced by iron supplements and pigmented beverages, creating a critical gap in evidence-based clinical guidance. Hence, the present in vitro study aimed to evaluate and compare the effectiveness of three different whitening modalities in removing extrinsic stains induced by iron supplements and pomegranate juice.

## Materials and Methods

**Figure 1:**
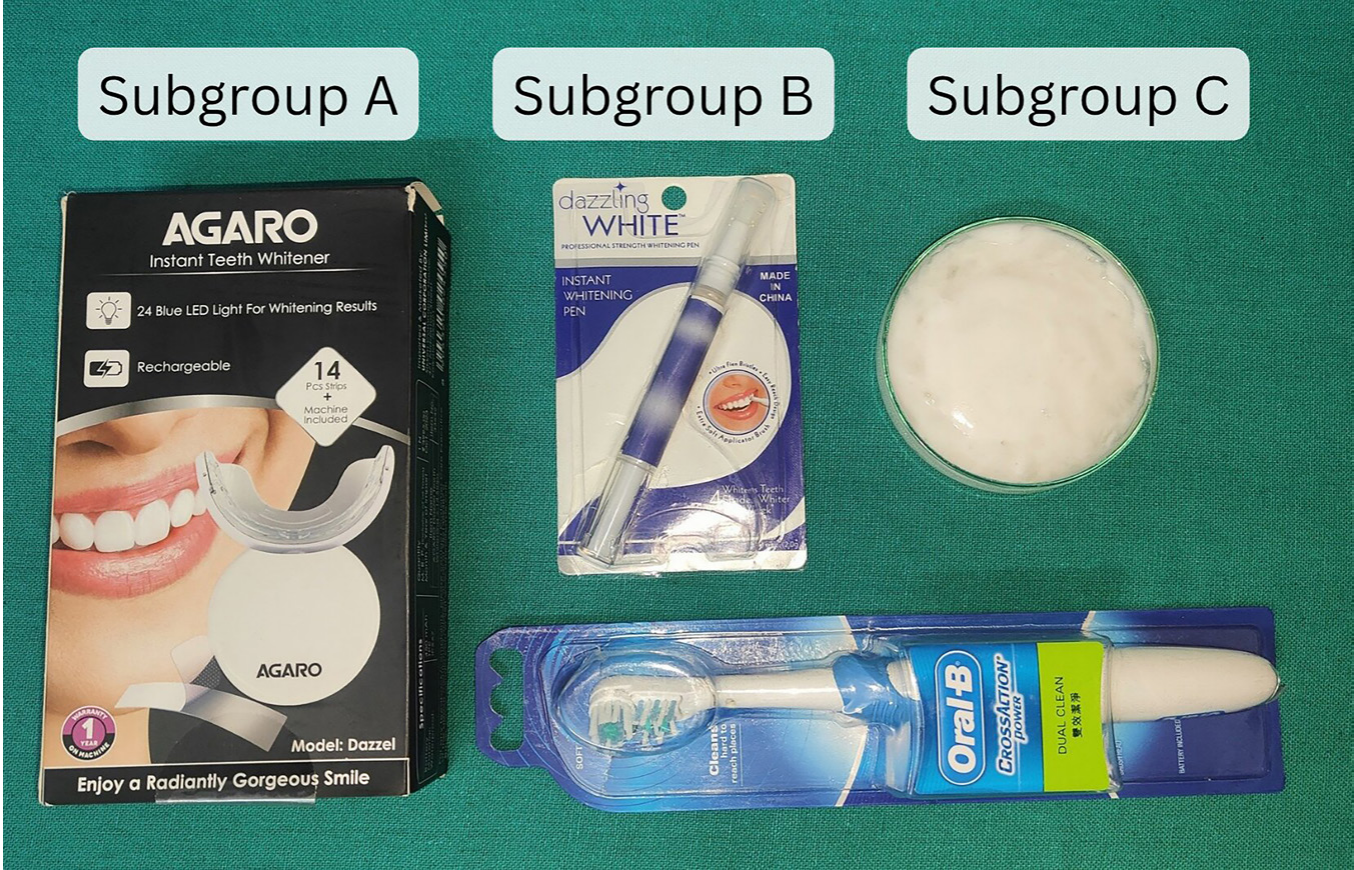
Whitening agents used in 3 subgroups.

**Figure 2:**
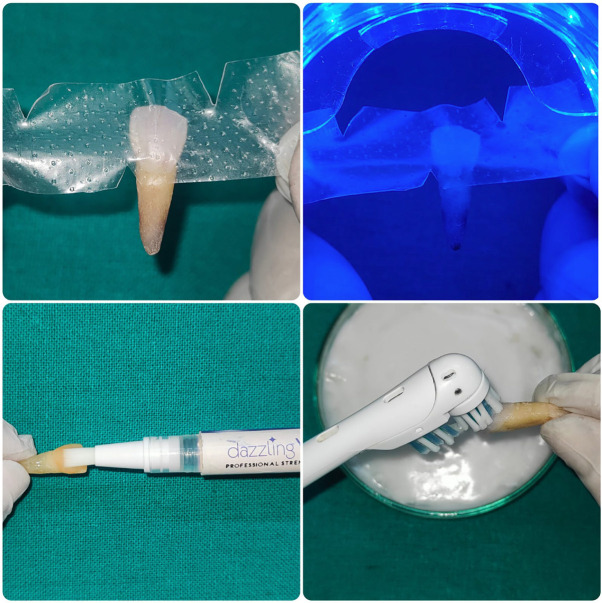
Application of whitening modalities.

This in vitro study was conducted in the Department of Pediatric and Preventive Dentistry, Institute of Dental Sciences, Bareilly (U.P.), in collaboration with B.I.U. College of Pharmacy, Bareilly, and I.T.S. Dental College, Murad Nagar, Uttar Pradesh. The sample size was calculated using G*Power 3.1 software (Franz Faul universitat, Keil, Germany). A total sample size of 60 specimens was estimated to achieve 80% power to detect a statistically significant difference at a significance level of 0.05 (7). Inclusion criteria a. Sound teeth with normal morphology Exclusion criteria a. Teeth exhibiting developmental defects, cracks or fractures b. Teeth with enamel discolouration c. Teeth with decalcifications d. Teeth with carious lesions RANDOMIZATION AND ALLOCATION: The specimens were randomly allocated into study groups using a computer-generated randomization sequence. STANDARDIZATION PROCEDURE: All samples were cleaned, and a standardized window was fabricated on the enamel surface to ensure uniform area exposure, after which they were stored in artificial saliva under controlled conditions. All procedures were performed by a single calibrated operator to eliminate inter-operator variability. Experimental conditions were standardized across all groups using a calibrated digital timer, while specimens were maintained at 37 ± 1 °C under controlled environmental conditions and stored in artificial saliva to ensure uniformity. SPECIMEN PREPARATION This study was approved by the Institutional Ethics Committee of the Institute of Dental Sciences, Bareilly (Approval No: IEC/289/2024/04). 60 permanent maxillary incisors were included in the study. All the specimens were cleaned using an ultrasonic scaler to remove any debris. The window on labial surface of the teeth was etched using Orikam Neoendo Neoetch Gel (37 % phosphoric Acid) for 60 s, and rinsed with distilled water for 30 s according to a protocol given by Meireles et al. (12) The teeth (60) were randomly assigned into two groups as follows: Group I: Iron medication staining group (n = 30) Group I.A: Tooth whitening strips (n = 10) Group I.B: Tooth whitening gel (n = 10) Group I.C: Customized herbal tooth whitening product (n = 10) Group II: Pomegranate juice staining group (n = 30) Group II.A: Tooth whitening strips (n = 10) Group II.B: Tooth whitening gel (n = 10) Group II.C: Customized herbal tooth whitening product (n = 10) STAINING PROCEDURE Group I: Iron medication staining: 10 ml of commercially available iron syrup (Hemfer New Kid Mango Flavour Syrup, ALKEM Alkem Laboratories Ltd., Mumbai) was diluted with artificial saliva to make up 250 ml solution and the total available iron content in the solution was 100 mg.(4) The samples were individually immersed in the iron solution for 5 minutes, twice daily, over a period of 15 consecutive days. The samples were then rinsed with distilled water and stored in artificial saliva. Group II: Pomegranate juice staining: The samples were immersed in freshly prepared pomegranate juice for 10 minutes, followed by rinsing with distilled water and stored in artificial saliva. This procedure was repeated daily for 15 consecutive days (13). WHITENING PROCEDURE (Figs. 1,2), (Table 1) 1.

1

2

Table 1

**Figure 3:**
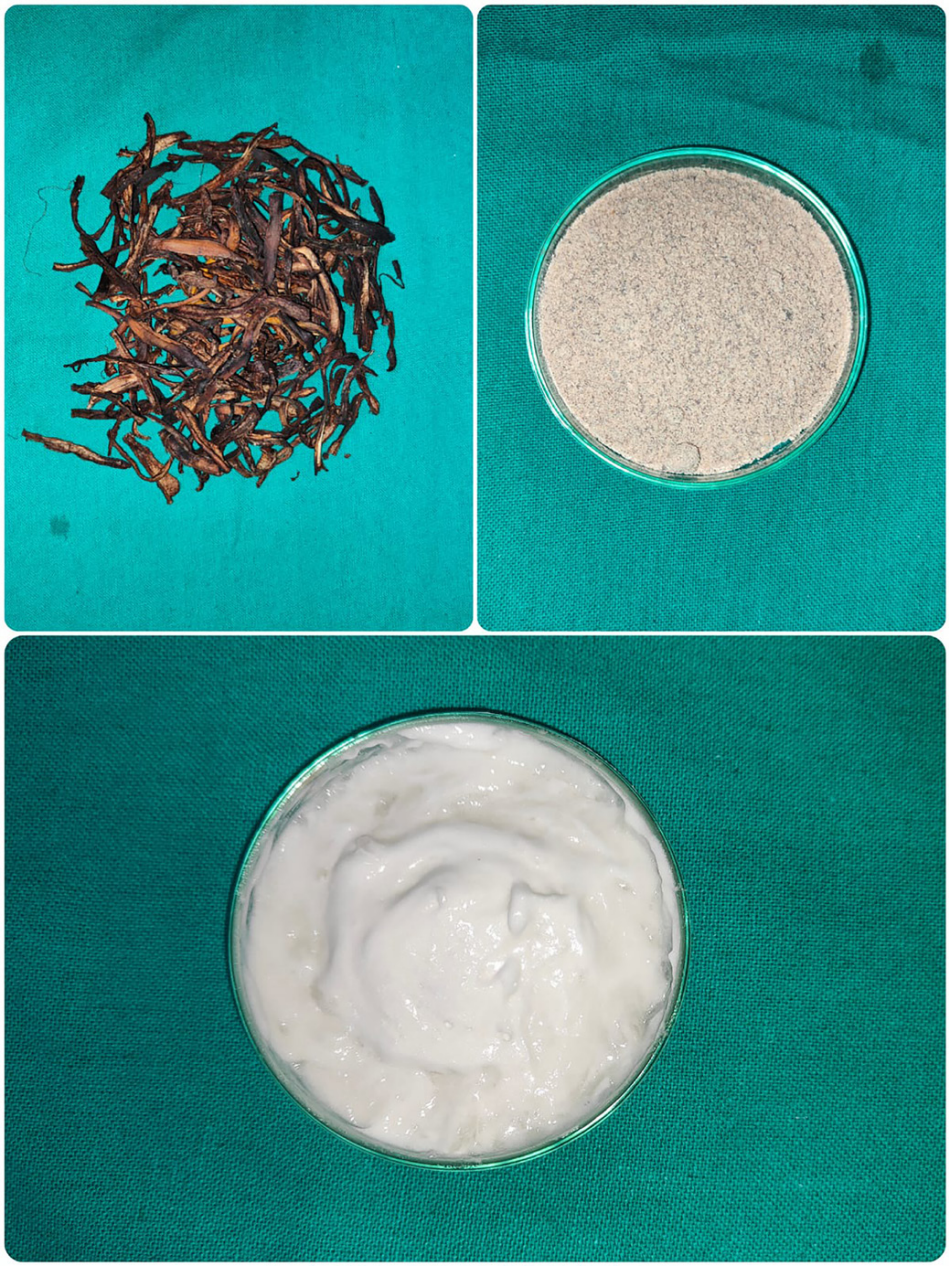
Preparation of herbal toothpaste.

Group I.A and Group II.A :AGARO Dazzle Instant Teeth Whitening Strips (Universal Corporation Ltd., Tianxiang Blvd, Jiangxi, China) were applied to the tooth surface, followed by exposure to LED light for 30 minutes once daily for 14 days. 2. Group I.B and Group II.B : White Drops Teeth Whitening Pen Gel was applied uniformly to the tooth surface using the applicator tip and allowed to remain on the tooth for 5 minutes once daily for 14 days. 3. Group I.C and group II.C: The customised herbal toothpaste was applied on the tooth surface using a powered toothbrush for 2 minutes twice daily for 14 days. Brushing protocol In subgroup C, a standardized brushing protocol was implemented, the specimens brushed for 120 seconds twice daily. Brushing was performed using an Oral-B® PRO CrossAction electric rechargeable toothbrush (Hi-P Housing Appliance Co., Ltd., Shanghai, China), which was held parallel to the sample to ensure consistency. Sample Preparation and procedure (Fig. 3).

3

The banana samples were collected, washed and air-dried. The dried samples were cut into small pieces, blended, and pulverized using a mortar and pestle. A 25 g portion of the powdered sample was soaked in 75 mL of 70% ethanol (1:3 ratio) in a conical flask, ensuring thorough mixing for effective extraction. The mixture was sealed and kept at room temperature for 72 hours with intermittent shaking to enhance extraction. Afterward, the extract was filtered using filter paper, and the filtrate was collected in clean conical flask (14). Preparation of herbal toothpaste All the ingredients mentioned in Table 2 (15) were mixed in a mortar and pestle, then triturated with precisely weighed glycerine until a semi solid substance was created.

Table 2

To adjust the consistency of the mixture, carboxymethyl cellulose was added and pepper mint oil was added for aroma. MEASUREMENT OF COLOUR CHANGE The shade of each sample was measured using a CS-600A/B Portable Colour Spectrophotometer (Hefei Chenchuang Instrument Co., Ltd., China). For each sample, three readings were obtained as follows: 1. The first reading was recorded at baseline (T0). 2. The second reading was recorded after 14 days of the staining procedure (T1). 3. The third reading was recorded after 14 days of the whitening procedure (T2). The CIE L*a*b* colour setup was used for colour analysis. The complete change in colour (E*) was determined using the following formula: E(L*a*b*) = [(L*) 2 + (a*) 2 + (b*) 2]1/2 where a* value is defined as the red-green axis, the b* value as the blue-yellow axis and the L* value defines lightness. L*, a* and b*.E between 3.7 and 1 represents clinically acceptable results (1). Statistical Analysis Data was entered in Microsoft excel spread sheet. Descriptive statistics like percentage and frequency distribution were calculated. Interferential statistics like ANOVA test were utilized for the purpose to discover statistical differences between 3 groups using SPSS software, version 21 (IBM corp. released 2011). P value less than 0.05 was considered statistically significant. The obtained data were statistically analysed using One-way ANOVA, Paired t-test, and Unpaired t-test to determine intra-group and inter-group differences in colour parameters.

## Results

**Figure 4:**
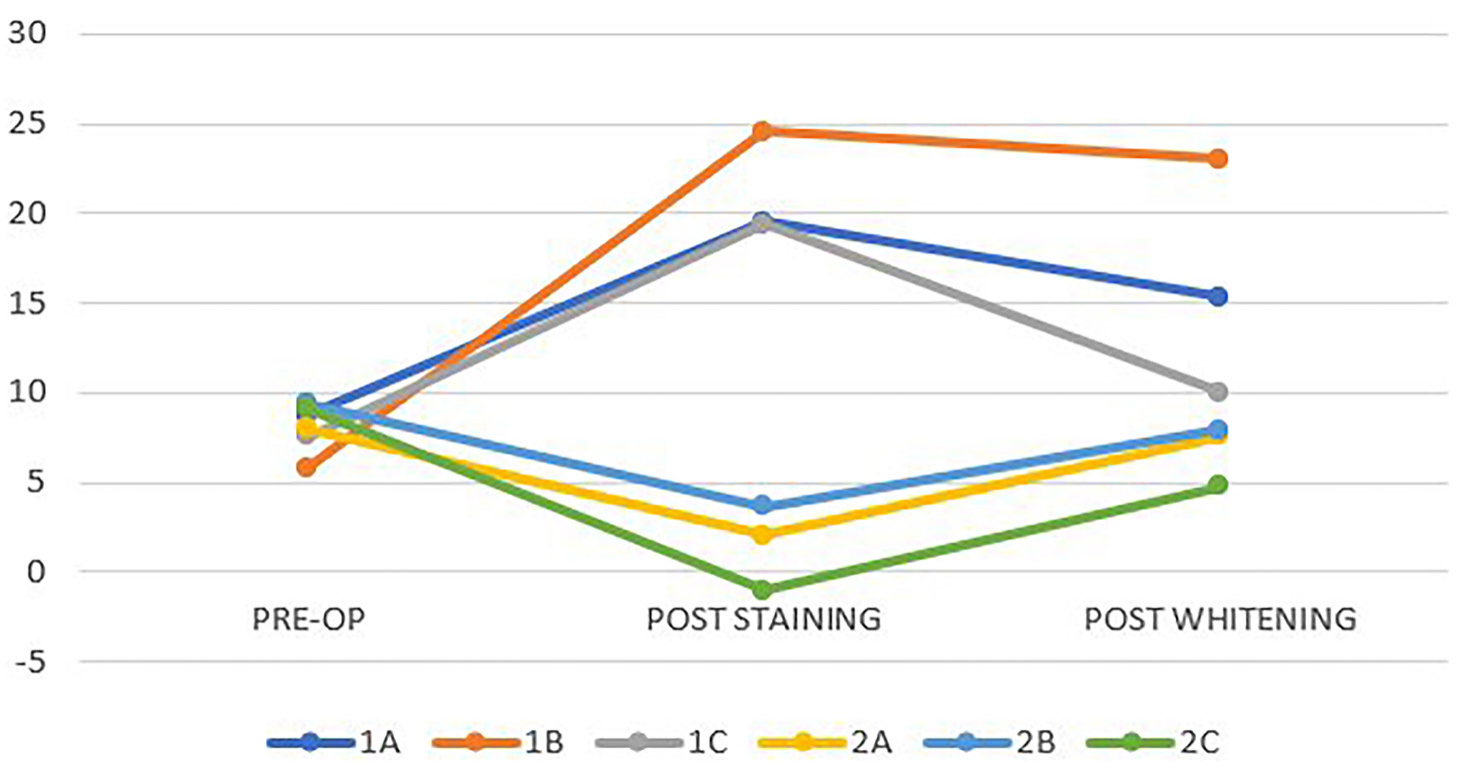
Graph shoring intra-group comparisons of colour change (ΔE) between post-staining and post-whitening phases.

Table 3 and Fig. 4 present intra-group comparisons of colour change (E) between post-staining and post-whitening phases using the paired t-test.

Table 3

4

A reduction in E values was observed across all groups following whitening. In iron-stained samples, Subgroup I.C showed the highest improvement (39.32%), followed by I.A (25.10%) and I.B (23.67%). In pomegranate-stained samples, Subgroup II.C demonstrated the greatest improvement (45.79%) (p &lt; 0.001) Table 4 compared colour parameters (L, a, b) Post whitening between Subgroups A, Subgroups B and Subgroups C which revealed statistically significant differences in a, b, and E values (p&lt;0.05), while L remained non-significant. Subgroup B retained a positive a* value (red shift) even after whitening, while subgroups A and C showed negative values. Subgroup B exhibited more persistent red discoloration.

Table 4

The higher b* values indicated that this subgroup had the highest level of residual yellow discoloration. Subgroup C showed the lowest yellow chromaticity (b* values). E values revealed that subgroup B had the highest residual colour while least residual colour was seen in subgroup C. Table 5 show the intra-group comparison of colour change (E) between the post-staining and post-whitening periods using the paired t-test. In Subgroup A (whitening strips), E decreased from 12.01 ± 6.00 to 8.75 ± 3.80, showing 27.14% improvement with a statistically significant difference (p = 0.0469).

Table 5

In Subgroup B (whitening pen gel), E reduced from 17.80 ± 6.23 to 13.963 ± 4.70, showing 21.57% improvement (p = 0.0342), indicating the least whitening effect among the groups.In Subgroup C (herbal formulation), E decreased from 15.349 ± 4.77 to 8.822 ± 3.08, showing the highest improvement (42.52%), which was highly significant (p = 0.00000878). Table 6 presents the comparison of colour change (E) at two different time points among the study groups using a paired t-test.

Table 6

Within-group analysis showed a statistically significant reduction in colour change after whitening in both the iron medication group (p = 0.00344) and the pomegranate juice group (p = 0.0000964). Notably, the pomegranate group demonstrated a greater whitening response, with lower residual colour change compared to the iron medication group.

## Discussion

Dental aesthetics have emerged as an essential component of contemporary oral healthcare, driven by an increasing emphasis on physical appearance, self-image, and overall psychosocial well-being in modern society (16). In this context, tooth discoloration-particularly extrinsic staining-represents a common aesthetic concern. Extrinsic stains occur due to dietary habits, with the deposition of chromogenic substances that adhere to enamel surface and produce visible colour changes (17). Consequently, the demand for aesthetic enhancement procedures has increased significantly. Iron-containing compounds are well known to induce extrinsic stains by interacting with the tooth surface and forming dark, insoluble deposits. The primary cause of discoloration is the chemical reaction between the iron in ferrous sulfate and the minerals present in teeth. Ferrous sulfate, in particular, can cause tooth discoloration that typically appears black or dark brown due to the formation of iron sulfide (18). Similarly, dietary pigments from beverages and foods such as tea, coffee, berries, pomegranate, and cola can also contribute to extrinsic staining (19). Tooth whitening dates back to when Haywood et al. reported that the tooth whitening effects of 10% carbamide peroxide, used for tray bleaching over a 6-month period lasted for up to 90 months. Since then, carbamide peroxide is widely used both in professional settings and through various OTC products for tooth whitening.(20) In our study we have used 2 OTC products i.e. teeth whitening strips,teeth whitening gel and a customized toothpaste. The samples were randomly divided into two groups based on the staining agent: iron medication solution and freshly prepared pomegranate juice. The results demonstrated a marked colour change following exposure to iron medication (E = 16.88 ± 6.89), (Table 6) indicating a strong staining potential on enamel surfaces. The pomegranate juice produced lower staining as compared to iron medications but produced a notable colour change (E = 13.22 ± 4.61). Overall, iron medication produced greater staining compared with pomegranate juice. This may be explained by the oxidation of iron ions forming ferric complexes that interact with phosphate groups in hydroxyapatite, resulting in insoluble dark deposits on the enamel surface. In contrast, natural pigments extracted from plants, fruits, and vegetables contain anthocyanins, responsible for red-purple pigmentation that produces organic stains (21). All recorded E values exceeded the clinically perceptible threshold, indicating that the observed colour changes were visually significant for both iron medications and pomegranate juice.Values reported by Nemtoi et al. (22) and Priya B et al. (23) reported notable colour change produced by pomegranate juice.These findings are consistent with those reported by Tayebi et al., (3), who also observed significant discolouration associated with iron-containing medications. Among the whitening modalities evaluated, the greatest colour improvement was observed with the herbal formulation (Subgroup C), followed by whitening strips (Subgroup A) and whitening pen gel (Subgroup B). Percentage improvements were 42.52% for the herbal formulation, 27.14% for whitening strips, and 21.57% for whitening pen gel, indicating effective removal of extrinsic stains (Table 5). The herbal formulation prepared from banana peel extract produced the highest colour recovery. Interest in natural tooth-whitening products has increased due to concerns regarding potential adverse effects of commercial bleaching agents. Also, potassium nitrate present in banana has desensitizing effects and also contributes to whitening potential. Magnesium salts may provide mild abrasive action (24). The whitening pen showed comparatively lower E value reduction in the present study, previous research has demonstrated favourable outcomes with certain paint-on systems. Bizhang et al. evaluated a gel containing peroxycaproic acid and observed a significant whitening effect even after a single application (25). Similarly, Tadin et al. found that 100% of participants using a whitening pen perceived a visible whitening effect (26). These studies demonstrated that whitening pens effectively reduce E values; however, they did not include a comparative evaluation with whitening strips or other OTC whitening agents. Kim YM et al. reported that both strip-type and paint-on-type OTC bleaching agents produced perceptible colour changes, with the strip-type group demonstrating greater mean E values (E=4.05) compared to the paint-on group (E=2.12), meeting ISO criteria for clinically perceptible whitening (E 2) the results are consistent with the findings of the present study (E &gt; 2). (8) Oliveira et al. also reported significant improvements in tooth lightness and reductions in yellowness following the use of hydrogen peroxide strips (27). Comparable findings were reported by Agarwal et al., who reported superior efficacy of strip systems compared to paint-on gels which could be attributed to the longer contact time and the presence of hydrogen peroxide in the strip. A statistically significant difference was detected between whitening gel (E=3.8 ± 3.12) and whitening strips (E=8.9 ± 3.64) (28). De Freitas et al., mentioned that the flexible plastic matrix allows better adaptation to tooth surfaces compared to paint-on gels, ensuring sustained contact and enhanced peroxide penetration. The relatively lower performance of the whitening pen observed in our study may be attributed to reduced contact time, uneven application, lower retention of the active agent, and limited sustained peroxide diffusion (29). Consistent with the previous research, our study demonstrated that although both modalities exhibited measurable whitening efficacy, differences were observed in the consistency of the results where whitening strips produced superior outcomes compared to gel formulations. On comparing the subgroups, the greatest whitening improvement was observed in Subgroup 2C, where pomegranate-stained teeth were treated with the herbal formulation. Conversely, the least whitening effect was observed in Subgroup 1B, where iron-stained specimens treated with the whitening pen gel exhibited the highest residual E (E=17.540 ±4.06) values. These differences may be attributed to the distinct mechanisms of iron- and pomegranate-induced discolouration. Iron ions bind strongly to phosphate groups in hydroxyapatite, forming stable inorganic deposits that are resistant to bleaching (4). In contrast, pomegranate stains are mainly organic in nature and are more susceptible to oxidative degradation. Similar findings were reported by Taneja et al., who observed greater bleaching efficacy for fruit-based stains than metallic stains (12). Overall, the results indicated that iron-containing solutions produced more intense staining, whereas pomegranate juice demonstrated comparatively greater colour reversibility following whitening procedures. The results indicate that although all whitening modalities reduced extrinsic discolouration, their effectiveness varied depending on the type of stain and delivery mechanism. Systems providing greater surface contact and prolonged exposure of the active agent produced more pronounced colour improvement. Recent studies have emphasized the role of delivery systems and active agent concentration in determining whitening efficacy. The superior performance of the herbal formulation observed in the present study may be attributed to its combined abrasive and phytochemical action, which facilitates stain removal. However, variability in outcomes across studies highlights the influence of experimental protocols, stain composition, and measurement techniques, warranting cautious interpretation. Limitations One of the primary limitations of the present study is its in vitro design. Laboratory conditions differ from the oral environment, where the continuous outward flow of dentinal fluid through dentinal tubules may limit the penetration of bleaching agents. Furthermore, factors such as salivary flow, oral biofilm, dietary habits, temperature variations, and masticatory forces cannot be fully replicated in vitro. Patient-related variables including brushing practices, compliance with whitening protocols, and variations in enamel composition may also influence clinical outcomes.

## Conclusions

Within the limitations of this in vitro study, both iron medication and pomegranate juice produced significant extrinsic staining, with iron demonstrating greater staining potential. Among the tested agents, the herbal formulation showed the highest whitening efficacy. However, these findings should be interpreted with caution, as in vitro conditions do not fully replicate the oral environment. Further in vivo studies are required to validate these results.

## Figures and Tables

**Table 1:** Comparison of Whitening Agents, Composition, and Application Regimen Across Study Groups.

GROUP CODE	WHITENING AGENT	COMPOSITION	APPLICATION REGIMEN
I.A and II.A	AGARO Dazzle Instant Teeth Whitening Kit	HP, glycerol, water, carboxymethyl carbomer, polyvinylpyrrolidone, sodium hydroxide , sodium saccharin, menthol, mint flavour	14 days application, once daily for 30 mins
I.B and II.B	Dazzling white, DR.Fresh Inc., Canada	HP, purified water, denatured alcohol, polyvinyl, pyrrolidone, polyethelene, glycol	14 days application, Once daily for 10 min
I.C and II.C	Herbal Toothpaste	Mentioned in Table 1	14 days application,Twice daily for 2 minutes

1

**Table 2:** Composition of Customised Toothpaste.

INGREDIENT	PROPERTY	QUANTITY
Calcium carbonate	Filler or abrasive	10
Glycerine	Humectant	10 ml
Sodium benzoate	Preventive	0.5 gm
Sodium lauryl sulphate	Foaming agent	1.5 gm
Sodium Saccharin	Sweetening agent	1 gm
Sodium CMC	Binder or thickening agent	0.5 gm
Banana peel extract	Active whitening ingredient	2 ml
Peppermint oil	Flavouring agent	q.s

2

**Table 3:** Intra group (subgroup) comparison of colour stability parameters (ΔE) between two different time periods of the study among various groups of the study assessed using Paired t Test.

Groups and subgroups	Post Staining (Mean ± SD )	Post Whitening (Mean ± SD )	% improvement in colour stability	p-value
1A	12.190± 6.09	9.130 ±4.21	25.10%	0.03*
1B	22.948± 3.49	17.540 ±4.06	23.67%	0.00*
1C	15.512± 6.01	9.412 ±2.60	39.32%	0.02*
2A	11.844± 6.22	8.378 ±3.52	29.26%	0.04*
2B	12.656± 3.33	10.386 ±1.37	17.93%	0.04*
2C	15.186± 3.44	8.232 ±3.54	45.79%	0.00*

SD: Standard deviation, ΔE: colour difference, p value* < 0.05 = significant

**Table 4:** Post whitening comparison of colour parameters (L,a,b) Post whitening between Subgroups A, Subgroups B and Subgroups C of the study using ANOVA Test.

Parameters	Subgroup A (Mean ± SD )	Subgroup B (Mean ± SD )	Subgroup C (Mean ± SD )	p-value
L	64.08±3.37	63.89±4.451	61.500±5.57	0.145
a	-0.72±1.93	0.862±2.59	-0.463±0.99	0.028
b	11.451±4.96	15.48± 8.40	7.390± 4.97	0.000747*
ΔE	8.754± 3.80	13.963± 4.70	8.822± 3.08	0.0000593*

SD: Standard deviation, ΔE: colour difference , p value* < 0.05 = significant

**Table 5:** Intra group comparison of colour parameters (ΔE) at Two different time periods of the study among various subgroups of the study using paired t test.

Groups	Post staining (Mean ± SD)	Post whitening (Mean ± SD)	% improvement in colour stability	p-value
Subgroup A	12.01±6.00	8.75±3.80	27.14%	0.0469*
Subgroup B	17.80±6.23	13.963± 4.70	21.57%	0.0342*
Subgroup C	15.349± 4.77	8.822± 3.08	42.52%	0.00000878*

SD: Standard deviation, ΔE: colour difference, p value* < 0.05 = significant

**Table 6:** Overall comparison of colour parameters (ΔE ) at Two different time periods of the study among various groups of the study using paired t test.

Groups	Post staining (Mean ± SD)	Post whitening (Mean ± SD)	% improvement in colour stability	p-value
Group 1	16.8833±6.89	12.027±5.33	28.76%	0.00344*
Group 2	13.2286±4.61	8.9986 ±3.05	32.03%	0.0000964*

SD: Standard deviation, ΔE: colour difference, p value* < 0.05 = significant
